# Differentiating Dengue from COVID-19: Comparison of Cases in Colombia

**DOI:** 10.4269/ajtmh.20-0912

**Published:** 2021-07-09

**Authors:** Fernando Rosso, Luis G. Parra-Lara, Olga L. Agudelo-Rojas, Diana M. Martinez-Ruiz

**Affiliations:** 1 Fundación Valle del Lili, Cali, Colombia;; 2 Universidad Icesi, Cali, Colombia

## Abstract

The differentiation between dengue and COVID-19 diagnoses is a challenge in tropical regions because of the similarity of symptoms and limited access to specific diagnostic tests for each disease. The objective of this study was to describe the initial symptoms and laboratory test values of patients who presented to the emergency department with dengue or COVID-19. A cross-sectional study was performed in a single center in Cali, Colombia. The inclusion criteria were patients with a diagnosis of dengue or COVID-19 who were older than 14 years of age. All patients experienced fever or other symptoms for fewer than 10 days. Linear regression was performed to evaluate the differences in the neutrophil-lymphocyte ratio (NLR) between patients diagnosed with COVID-19 and dengue, and was adjusted for sex and age group (≤ 31 and > 31 years). The sample size was calculated to test the hypothesis that the median NLR in COVID-19 patients is higher than that in dengue patients. A *P* value < 0.05 was considered statistically significant for all analyses. A total of 93 patients were included: 70 with dengue and 23 with COVID-19. Dengue patients were younger than COVID-19 patients. There were significant differences between dengue and COVID-19 patients regarding platelet count (*P* < 0.01), neutrophil count (*P* < 0.01), NLR (*P* < 0.01), and abnormal alanine transaminase (ALT) (*P* = 0.03). The NLR was significantly higher in COVID-19 patients than in dengue patients (*P* < 0.01). In conclusion, during the first week of symptoms, absolute neutrophil count, NLR, and platelet count could help guide the initial differential approach between dengue and COVID-19. These findings could be useful in geographical areas with a lack of resources.

## INTRODUCTION

In tropical countries, dengue virus infection is a public health problem because of the hyperendemicity of four virus serotypes and their effects on human health.^1^ During the first 4 weeks of 2020, in the region of the Americas, 125,514 dengue cases were reported (12.86 cases per 100,000 population), including 27 deaths and 498 severe dengue cases (0.4%).^2^ In addition, SARS-CoV-2, the etiologic agent of COVID-19, was declared as a pandemic agent in March 2020 by the World Health Organization (WHO) because of its rapid spread worldwide, and approximately 520,000 deaths were reported through June 2020.^3^

During the COVID-19 pandemic, the coexistence of COVID-19 with other diseases is not unusual, and misdiagnoses may be inevitable. These issues are more common in tropical countries affected by other infectious diseases, such as dengue, that cause signs and symptoms similar to COVID-19.^4^^–^^8^ Major challenges in diagnosis often occur because of the limited access to specific diagnostic tests for each disease^9^^,^^10^ and a misdiagnosis in the acute stage of these diseases can lead to an incorrect therapeutic approach and consequently to unfavorable clinical outcomes for patients.

Complete blood count (CBC) is the single most common test performed in patients.^11^ In dengue, the CBC is characterized by progressive leukopenia followed by a rapid decrease in the platelet count in conjunction with a rising hematocrit compared with the baseline hematocrit, and these CBC abnormalities usually precede the plasma leakage/critical phase of the disease.^7^^,^^12^ In contrast, patients with COVID-19 may present with leukopenia or leukocytosis, eosinopenia, and lymphopenia, and lymphopenia is the most common abnormality, which makes it a useful and reliable indicator of the severity of the disease.^13^ Other CBC parameters, such as the neutrophil-lymphocyte ratio (NLR), have been described as independent biomarkers for indicating poor clinical outcomes in COVID-19 patients.^14^

Aminotransferases are used to assess organ involvement, such as hepatocellular lesions, in severe dengue,^15^ and in COVID-19, they are closely related to the severity of the disease and prognosis.^16^ In low- and middle-income countries, access to specific diagnostic tests and the funding for them is limited. For this reason, commonly implemented laboratory tests, such as the CBC and biochemical tests, should be evaluated as the potential tests to differentiate between dengue and COVID-19.

The main objective of this study is to evaluate the value of CBC results in the differentiation between dengue and COVID-19 diagnoses. Secondly, the study also aims to compare the clinical presentations of both diseases.

## MATERIALS AND METHODS

### Setting and data source.

A cross-sectional study was performed at the Fundación Valle del Lili (FVL) in Cali, Colombia. FVL is a nonprofit university hospital that serves as a referral facility for the southwestern region of Colombia. The incidence rate of dengue in Cali was 147.5 cases per 100,000 during March 2020.^17^ On May 31, 2020, the Instituto Nacional de Salud (INS) estimated an R0 of 2.28 (95% CI 2.06–2.52), a lethality of all cases at 1.14% (95% CI 1.00–1.30%), and a total of 332,801 symptomatic cases for the city.^18^ At the time of the study, the city had two outbreaks: dengue and COVID-19.^19^

#### Ethics statement.

The Institutional Review Board – Comité de Ética en Investigación Biomédica at FVL approved this study (IRB/EC No. 919), and it was conducted after the Declaration of Helsinki and Resolution 8430/1993 from Colombia Ministry of Health. Informed consent was not required because this study was performed retrospectively from two secondary Microbiology Laboratory and Epidemiological Surveillance Committee databases.

### Patients and data.

We included two different groups of patients: dengue and COVID-19 patients. In both groups, the inclusion criteria were as follows: patients were older than 14 years of age, arrived at the ER with less than 10 days of fever or other symptoms (i.e., asthenia/adynamia, myalgia, diarrhea, headache, intense abdominal pain, arthralgia, nausea, or rash) and were eventually diagnosed with dengue or SARS-CoV-2 between March 1, 2020 and May 4, 2020. All dengue patients had positive NS1 or positive IgM, and all dengue patients had confirmed seroconversion.

We collected demographic, clinical, and CBC data for the first CBC performed during care in all patients. All data were obtained directly from the clinical charts and other databases. The selection of symptoms was made based on the WHO Dengue Guidelines^20^ for dengue and the descriptions previously reported in the literature for COVID-19.^8^^,^^21^

A decrease in hemoglobin was defined as hemoglobin less than 10 g/dL, and oxygen support was defined as any method that increased the fraction of inspired oxygen (FiO2) in patients, such as a nasal cannula, mask, high-flow cannula, or nonrebreathing mask.

### Diagnostic tests.

Dengue cases were diagnosed through blood samples using the SD BIOLINE Dengue DUO^®^ NS1 Ag + Ab Combo test (Alere Inc., Waltham, MA). Seroconversion was defined as patients who were positive for anti-DENV antibodies, positive for either anti-DENV IgG or IgM from the acute phase to convalescent phase or positive for NS1.

According to the infectious diseases specialist’s criteria who handled each case, a nested RT-PCR DENV 1-4 or seroconversion evaluation was conducted in patients with suspected coinfection between both viruses. The nested PCR DENV 1-4 was performed at the Virology Laboratory, Universidad del Valle, Cali, Colombia, and this was in-house laboratory test based on the protocol of Lanciotti et al.^22^

COVID-19 patients were diagnosed with nasopharyngeal swabs using the CDC 2019-nCoV Real-Time RT-PCR Diagnostic Panel protocol (CDC, Atlanta, GA) or the VIASURE^®^ SARS-CoV-2 Real-Time PCR Detection Kit (Certest Biotec S.L., Zaragoza, Spain). Our hospital implemented the protocol developed by the Charité Institute of Virology-Universitatsmedizin Berlin, Alemania, recommended by the WHO.^23^

### Study size.

The sample size was calculated to test the hypothesis that the median NLRs in COVID-19 patients is higher than that in dengue patients. For a confidence level of 95% and a power of 80%, the sample size was for each group^24^:

$$n=\frac{\left[\log\left(\frac{1}{2}+\sqrt{\frac{1}{4}+{\left(\frac{\varphi_{1}}{m_{1}}\right)}^{2}}\right)+\log\left(\frac{1}{2}+\sqrt{\frac{1}{4}+{\left(\frac{\varphi_{2}}{m_{2}}\right)}^{2}}\right)\right]{\left(z_{\alpha /m}+z_{\beta }\right)}^{2}}{{\left(\log\left(m_{1}\right)-\text{log}(m_{2})\right)}^{2}}=23$$

where

$z_{\alpha /m}=-2.13$ is the *z* value of the significance level adjusted by Bonferroni (*m* = 6 comparisons: Dengue versus COVID-19 stratified by intensive care unit [ICU], sex, age group, and interaction).

$m_{1}=4.7$ is the median NLR in COVID-19 patients.^25^

$\varphi_{1}=4.68$ is the standard deviation of NLR in COVID-19 patients.^26^

$m_{2}=0.90$ is the median NLR in dengue patients (obtained from a previous dengue study published by our group.^27^

$\varphi_{2}=6.85$ is the standard deviation of NLR in dengue patients (obtained from a previous dengue study published by our group.^27^

With this sample size, it was possible to identify increases greater than 3.8 in the NLR in COVID-19 patients compared with dengue patients. However, we decided to include all the cases found in the study period because we attempted to answer a question that has not been previously resolved in the literature.

### Statistical analysis.

Continuous variables are presented as the median and interquartile range or mean and standard deviation. Qualitative variables are presented as absolute frequencies and percentages. Qualitative variables were compared by the chi-square test or Fisher’s exact test as appropriate. Continuous variables were compared using the Mann–Whitney *U* test for right-tailed hypothesis tests, with the assumption that CBC parameters are higher in COVID-19 patients, and a left-tailed hypothesis test was used for transaminases in COVID-19 patients.

Linear regression was performed to evaluate the difference in the NLR logarithmic transformation in patients with dengue and COVID-19, adjusted by sex and age group (≤ 31 and > 31 years, according to distribution by quartiles), and then a *post hoc* Tukey test was used to identify differences in adjusted NLR means. Additionally, to evaluate the NLR classification capacity of COVID-19 compared with dengue, the area under the ROC curve was calculated using the optimal cutoff point obtained with the Youden method.

An analysis stratified by severity (defined as ICU admission) was performed for CBC parameters under the same hypothesis. A 5% significance level was applied. All analyses were performed using STATA^®^ (Version 14.0, StataCorp LP, College Station, TX).

## RESULTS

From a total of 123 patients diagnosed with COVID-19 and 183 patients diagnosed with dengue, 23 and 70 were included in each group, respectively, after meeting the inclusion and exclusion criteria. In the dengue patients, 38 cases had positive anti-DENV antibodies and 32 had positive anti-DENV antibodies and NS1antigen.

There were two cases of dengue and COVID-19 coinfection. The first case was confirmed by nested RT-PCR for DENV 1-4, and the second was confirmed by seroconversion (persistence of positive anti-DENV antibodies 24 days after onset of symptoms).

Seventy-one percent of the dengue patients were classified as dengue with warning signs, 17% as dengue without warning signs, and 12% as severe dengue. The most frequent warning signs were persistent vomiting (22.7%), bleeding manifestations (20%), and increased hematocrit (19.7%). The median length of hospital stay for COVID-19 patients was 8 days (IQR = 4–25 days), and for dengue patients, it was 3 days (IQR = 2–4 days).

Table 1 shows the demographics and clinical and laboratory findings for both diseases. Dengue patients were significantly younger than COVID-19 patients (*P* < 0.01). A lower median oxygen saturation at admission was reported in COVID-19 patients. Fever, myalgias, headache, and nausea were more frequent in dengue patients. The mean length of time of symptoms before consulting was significantly higher in COVID-19 patients (*P* = 0.017). Cough and dyspnea were more frequent in COVID-19 patients. There was less oxygen saturation in COVID-19 patients than in dengue patients (*P* < 0.001). A decrease in the hemoglobin value (< 10 g/dL) was more frequent in COVID-19 patients. There were no COVID-19 patients with leukopenia, and two patients had thrombocytopenia.

**Table 1:** Demographics and clinical and laboratory characteristics of COVID-19 and dengue patients (*N* = 93)

	COVID-19, *N* = 23	Dengue, *N* = 70
Characteristics	*n* (%)	*n* (%)
Demographics		
Median age (IQR)–yr	45 (32–62)	25 (17–42)
Male sex	12 (52.17)	34 (48.57)
Clinical		
Mean length of symptoms before consultation (SD)–days	6.04 (0.601)	4.29 (0.214)
Median of oxygen saturation at admission (IQR)–%	95 (89–98)	98 (96–99)
Cough	23 (100)	3 (4.29)
Fever	18 (78.26)	69 (98.6)
Dyspnea	14 (60.87)	1 (1.43)
Myalgia	10 (43.5)	60 (85.7)
Diarrhea	5 (21.7)	19 (27.1)
Headache	6 (26.1)	49 (70)
Intense abdominal pain	0	25/66 (37.9)
Arthralgia	0	56 (80)
Nauseas	3 (13.04)	32 (45.7)
Rash	0	22 (31.4)
Drop in hemoglobin (< 10 g/dL)	2 (8.7)	2 (2.9)
Respiratory support	5 (21.74)	1 (1.43)
Laboratory		
Leukopenia (leukocytes < 3,500)	0	26 (37.1)
Platelets < 150.000/uL	2 (8.7)	54 (77.1)
Platelets < 50.000/uL	0 (0)	21 (30)
AST > 2 times upper limit	4/8 (50)	41/62 (66.1)
AST > 3 times upper limit	0	34/62 (54.8)
ALT > 2 times upper limit	2/8^25^	38/63 (60.3)
ALT > 3 times upper limit	2/8^25^	24/63 (38.1)
Serum creatinine elevation from baseline, *N* = 49	0	7/49 (14.3)

ALT = alanine transaminase; AST = aspartate transaminase; IQR = interquartile range; SD = standard deviation.

The comparisons of CBC and aminotransferase results at ER admission are shown in Table 2. The white blood cell count was significantly higher in COVID-19 patients (*P* = 0.03), and there were also differences in neutrophil count (*P* < 0.01), NLR (*P* < 0.01), and platelet count (*P* < 0.01). Although the values of lymphocytes were not significantly higher in COVID-19 patients, both transaminase levels (alanine transaminase [ALT] and aspartate transaminase [AST]) were lower in these patients.

**Table 2:** Comparison between COVID-19 and dengue patients with respect to laboratory test values at ER admission (*N* = 93)

Laboratory test values	COVID-19, *N* = 23	Dengue, *N* = 70	*P* value
Median (IQR)			
White blood cell count–cells/µL	8,190 (5,280 – 10,420)	4,475 (3,090 – 6,870)	0.03
Neutrophil count–cells/µL	5,710 (3,500 – 8,090)	1,995 (1,360 – 3,400)	0.00
Lymphocyte count–cells/µL	1,330 (850 – 1,660)	1,270 (800 – 2,490)	0.81
NLR	4.01 (2.03–11.96)	1.38 (0.58–3.74)	0.00
Platelet count–×10^3^ cells/µL	244 (180–369)	84 (47–131)	0.00
ALT–IU per liter*	37.2 (26.45–114.38)	119.1 (38.1–212.3)	0.03
AST–IU per liter†	63.6 (33.15–103.05)	162.55 (53.7–311.2)	0.02

ALT = alanine transaminase; AST = aspartate transaminase; ER = emergency room; NLR = neutrophils-lymphocytes ratio; IQR = interquartile range.

* ALT: COVID-19 (*N* = 8) and dengue (*N* = 63).

† AST: COVID-19 (*N* = 8) and y dengue (*N* = 62).

**Figure 1.**
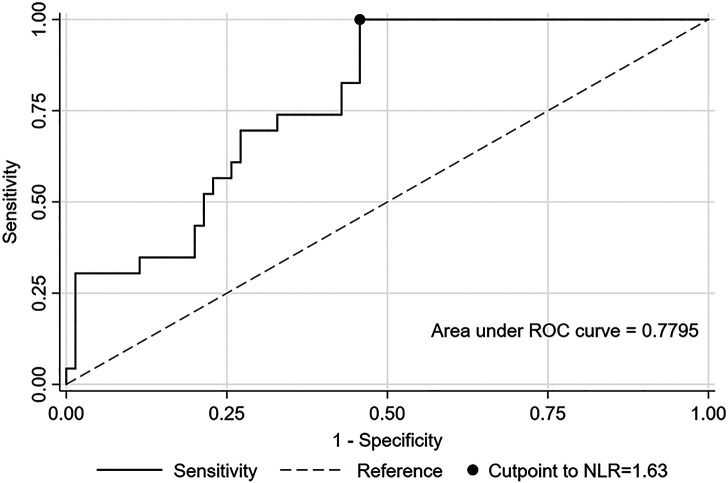
Receiver operating characteristic (ROC) curve of the predictive ability to classify between COVID-19 and dengue.

Table 3 shows that the NLR increased significantly in COVID-19 patients compared with dengue patients (*P* < 0.01). Predictive ability analysis showed that an NLR of 1.63 has an AUC of 77%, sensitivity of 100%, and specificity of 54% in the classification between dengue and COVID-19 (Figure 1).

**Table 3:** Linear regression for the NLR logarithm adjusted by sex, age, and diagnosis of COVID-19 vs. dengue

Variable	Coefficient	95% CI	*P* value
Intercept	0.2263	(−0.1416319; 0.5942426)	0.23
COVID-19	0.9094	(0.3902711; 1.4284385)	0.00*
Male sex	−0.2023	(−0.6237089; 0.2191761)	0.35
Age < 31 years	0.7338	(0.2852224; 1.1824053)	0.00

CI = confidence interval; NLR = neutrophils-lymphocytes ratio.

* *P* value adjusted by post hoc Tukey test.

The comparison of laboratory test values at emergency room (ER) admission stratified by severity is shown in Table 4. Twenty-nine patients were managed in the ICU: sixteen had COVID-19 and thirteen had dengue. The white blood cell count, neutrophil count, and NLR were significantly higher in COVID-19 patients than in dengue patients. In patients who were not admitted to the ICU, the neutrophil and platelet counts were significantly higher in COVID-19 patients. There were no dengue patients with respiratory distress syndrome.

**Table 4:** Comparison of laboratory test values at ER admission between COVID-19 and dengue patients by severity (defined as ICU admission)

	Non-ICU management, *N* = 64			ICU management, *N* = 29		
Variables	COVID-19, *N* = 7	Dengue, *N* = 57	*P*	COVID-19, *N* = 16	Dengue, *N* = 13	*P*
Median (IQR)						
White blood cell count–cells/µL	5,280 (4,670–5,950)	3,940 (3,000–6,330)	0.09	8,860 (7,220–12,790)	6,020 (4,460–7,480)	0.00
Neutrophil count–cells/µL	3,070 (2,610–4,080)	1,940 (1,270–3,330)	0.02	6,450 (4,835–10,935)	2,050 (1,610–3,750)	0.00
Lymphocyte count–cells/µL	1,660 (1,130–1,730)	1,300 (800–2,380)	0.33	1,235 (650–1,465)	1,090 (840–3,240)	0.84
NLR	1.84 (1.72–3.61)	1.39 (0.64–3.22)	0.16	5.09 (3.83–13.08)	1.33 (0.48–5.7)	0.01
Platelet count–×10^3^ cells/µL	193 (156–369)	94 (63–140)	0.00	257 (219.5–383.5)	24 (13–83)	0.08
ALT–IU per liter	52.9 (36–69.9)	123.9 (40.1–223)	0.14	36.2 (18.9–158.9)	106.5 (37.8–152.7)	0.14
AST–IU per liter	59.85 (32.3–87.4)	157.1 (53.7–315.5)	0.13	63.6 (34–118.7)	168 (78.9–245.3)	0.05

ALT = alanine transaminase; AST = aspartate transaminase; ER = emergency room; ICU = intensive care unit; IQR = interquartile range; NLR = neutrophils-lymphocytes ratio.

## DISCUSSION

Here we describe the symptoms and laboratory findings of patients who arrived at the ER diagnosed with dengue or COVID-19 at the time of both epidemics. Three key points can be extracted from our observations: 1) patients with dengue are mainly younger patients; 2) laboratory test values such as CBC and ALT are useful in the initial diagnostic approach; and 3) NLR predicts a diagnosis of COVID-19 versus dengue. These results might be useful in dengue hyperendemic areas and tropical regions where the initial differentiation between COVID-19 and dengue in their early stages can be challenging in some cases. These results may also help in clinical decision-making and prevent the misdiagnosis of dengue and COVID-19.

We found that dengue patients were younger than those with COVID-19. Dengue fever has been known to occur in both children and adults, but severe dengue occurs more commonly in older children.^28^ However, severe dengue in older adult patients has also been reported in tropical countries, and the mortality is increased in this population^29^^–^^32^ despite being only a minority of patients.^9^ In contrast, COVID-19 tends to affect adults and older adults, but the most severe cases affected are those over 60 years of age.^33^ Older adults above age 80 have a case fatality rate of 14.8%.^34^

Based on the symptoms at presentation, fever, rash, and intense abdominal pain were more frequent in dengue patients than in COVID-19 patients, who mostly had cough and dyspnea. COVID-induced rashes and abdominal pain were previously described,^35^^–^^37^ but these findings were not common in our population.

The NLR is an established inflammation marker that reflects systemic inflammation in critical care patients and has been used to assess both infectious and noninfectious diseases.^38^ The increase in NLR could indicate a poor clinical prognosis and could be an independent indicator of mortality.^14^^,^^39^ Dengue patients have a lower NLR compared with COVID-19. It is known that an elevated NLR is an independent prognostic biomarker that affects pneumonia progression in COVID-19 patients. The above can be explained by the increase in the neutrophil count and a lower limit of lymphocyte count in COVID-19 patients, whereas dengue patients have leukopenia with neutropenia but present with lymphocytosis (Table 2). Additionally, the NLR performed well for discriminating between COVID-19 and dengue in patients under the age of 31 years. We propose integrating the NLR in the diagnosis of COVID-19 in tropical regions, and future studies should be conducted to further evaluate the impact of the NLR on predicting severe cases or mortality.

Thrombocytopenia is common in critically ill patients and generally suggests organic dysfunction or physiological decompensation.^40^ In dengue, it coincides with defervescence (approximately 3–5 days) and the onset of hemorrhagic manifestations during severe infection (critical phase of the disease), which constitutes severe dengue.^20^ However, in COVID-19 patients, it can occur later in the clinical course with other symptoms of severity. It is speculated that thrombocytopenia can occur by four mechanisms: cytokine storm, direct infection of hematopoietic and bone marrow stromal cells, increased antibodies and immune complexes, and lung injury.^41^

Elevated aminotransferase levels were found in dengue patients. Aminotransferases are enzymes that catalyze transamination reactions and are markers of hepatocellular injury.^42^ In dengue, marked elevation constitutes one of the signs of disease severity since the liver is one of the target organs of virus infection,^43^ and these tests are routinely requested in patients hospitalized for dengue for clinical follow-up. In COVID-19, aminotransferases may be normal or slightly elevated; however, a marked elevation of these enzymes may constitute a marker of severity in the disease due to a systemic inflammatory response mechanism.^44^ Consequently, the dengue and SARS-CoV-2 viruses can affect the liver, and aminotransferase levels could be elevated in both diseases. Initially, they will be elevated mainly in dengue.

Dengue diagnosis is performed in tropical regions through characteristic symptom identification, such as fever, and laboratory findings, such as thrombocytopenia and capillary leakage.^20^ Currently, serological tests have been implemented and are used to confirm dengue virus infection. Nevertheless, the similarity in some of the symptoms between dengue and COVID-19, together with the potential cross-reactions in serological tests, could lead to difficulty in diagnosis and is an important issue to be considered. Several studies have reported false-positive dengue serology results in COVID-19 patients.^45^ In these patients, dengue virus infection is ruled out after controlled serological tests showing no evidence of seroconversion or persistence of positivity and the exclusion of the diagnosis of dengue by RT-PCR. Therefore, positive results in dengue serology should be carefully interpreted after the consideration of the possibility of COVID-19 patients.^46^

Finally, in the context of the SARS-CoV-2 pandemic in dengue hyperendemic regions, we recommend the careful evaluation of CBC and aminotransferase values, especially in regards to white blood cell count, platelet count, and NLR, for the evaluation of differential diagnoses during patient work up in the ER, and these values could be helpful in areas with limited resources and laboratory technologies.

### Limitations.

Our study is not without limitations, and the results should be interpreted in the context of a cross-sectional study. First, because our hospital provides health services to patients with advanced and complicated diseases, our study was prone to selection bias in this regard. Second, the data about the clinical characteristics of the patients were obtained directly from the clinical records and secondary databases; therefore, information bias could be introduced regarding the symptoms and the clinical presentation of the patients.

Third, the evaluation of the laboratory tests was only conducted upon admission to the hospital, and these laboratory parameters were not followed up during hospitalization. Fourth, serological tests were used to diagnose dengue, which has a lower performance than the RT-PCR method, which increases the proportion of false negatives. However, dengue clinical practice guidelines (CPG) in Colombia recommend its use and implementation.^47^ Fifth, two laboratory tests were used for the diagnosis of COVID-19 in the study because, in Colombia, the implementation of the diagnostic tests was initially the responsibility of the National Institutes of Health and later was the decision of the different hospitals and laboratories, according to the test availability. Finally, using serological tests for the diagnosis of dengue while using molecules for the diagnosis of COVID-19 reduced the diagnostic accuracy since a positive serological test does not always preclude acute infection.

## CONCLUSION

During the first week of symptoms, neutrophil and lymphocyte count, NLRs, and thrombocytopenia could guide the initial differential approach between dengue and COVID-19 patients. Thrombocytopenia tends to occur earlier in dengue patients and will present without fever. COVID-19 patients usually will have low platelet counts later in their clinical course and present with fever. Dengue patients tend to be younger at admission to the ER. These findings can be helpful for the differential diagnosis of both diseases in regions with a lack of resources.
